# *catena*-Poly[[[(2,2′-bipyrid­yl)copper(II)]-μ-l-alaninato] perchlorate monohydrate]

**DOI:** 10.1107/S1600536808040725

**Published:** 2008-12-13

**Authors:** Mircea Braban, Ionel Haiduc, Peter Lönnecke

**Affiliations:** a Facultatea de Chimie si Inginerie Chimica, Universitatea Babes Bolyai, Str. Arany Janos nr. 11, RO-400028 Cluj-Napoca, Romania; b Institut für Chemie und Mineralogie, Universität Leipzig, Johannisallee 29, D-04103 Leipzig, Germany

## Abstract

In the structure of the polymeric title complex, {[Cu(C_3_H_6_NO_2_)(C_10_H_8_N_2_)]ClO_4_·H_2_O}_*n*_, the carboxyl­ate group of the chelating amino acid is further linked to a neighbouring Cu centre, generating a supra­molecular single-stranded chain parallel to [010]. The structure displays inter­molecular N—H⋯O and O—H⋯O hydrogen bonding, which consolidates the crystal packing.

## Related literature

For related structures, see: Antolini *et al.* (1983 ▶); Masuda *et al.* (1991 ▶); Sgarabotto *et al.* (1999 ▶); Solans *et al.* (1992 ▶).
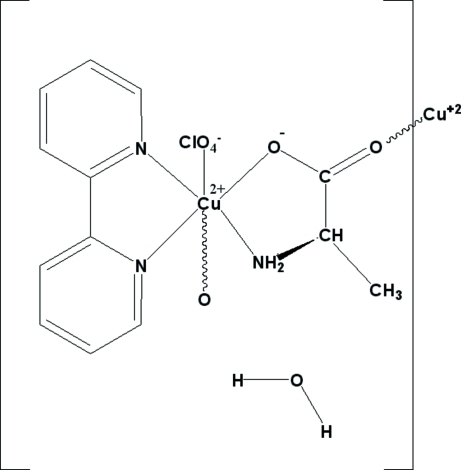


## Experimental

### 

#### Crystal data

[Cu(C_3_H_6_NO_2_)(C_10_H_8_N_2_)]ClO_4_·H_2_O
*M* *_r_* = 425.28
Monoclinic, 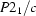

*a* = 13.1807 (10) Å
*b* = 8.2656 (6) Å
*c* = 16.1195 (13) Å
β = 110.606 (2)°
*V* = 1643.8 (2) Å^3^
*Z* = 4
Mo *K*α radiation
μ = 1.53 mm^−1^
*T* = 220 (2) K
0.60 × 0.30 × 0.30 mm

#### Data collection

Bruker SMART CCD area-detector diffractometer
Absorption correction: multi-scan (*SADABS*; Sheldrick, 1997 ▶) *T*_min_ = 0.460, *T*_max_ = 0.656
13671 measured reflections
3939 independent reflections
3611 reflections with *I* > 2σ(*I*)
*R*_int_ = 0.020

#### Refinement

*R*[*F* ^2^ > 2σ(*F* ^2^)] = 0.033
*wR*(*F* ^2^) = 0.078
*S* = 1.11
3939 reflections
290 parameters
All H-atom parameters refined
Δρ_max_ = 0.89 e Å^−3^
Δρ_min_ = −0.43 e Å^−3^

### 

Data collection: *SMART* (Bruker, 1998 ▶); cell refinement: *SAINT* (Bruker, 1998 ▶); data reduction: *SAINT*; program(s) used to solve structure: *SHELXS97* (Sheldrick, 2008 ▶); program(s) used to refine structure: *SHELXL97* (Sheldrick, 2008 ▶); molecular graphics: *ORTEP-3 for Windows* (Farrugia, 1997 ▶) and *DIAMOND* (Brandenburg, 1999 ▶); software used to prepare material for publication: *WinGX* (Farrugia, 1999 ▶).

## Supplementary Material

Crystal structure: contains datablocks I, global. DOI: 10.1107/S1600536808040725/tk2300sup1.cif

Structure factors: contains datablocks I. DOI: 10.1107/S1600536808040725/tk2300Isup2.hkl

Additional supplementary materials:  crystallographic information; 3D view; checkCIF report

## Figures and Tables

**Table 1:** Hydrogen-bond geometry (Å, °)

*D*—H⋯*A*	*D*—H	H⋯*A*	*D*⋯*A*	*D*—H⋯*A*
N3—H2*N*3⋯O7^i^	0.74 (4)	2.60 (4)	3.293 (4)	159 (5)
N3—H1*N*3⋯O1^ii^	0.83 (4)	2.48 (4)	3.225 (3)	149 (3)
N3—H1*N*3⋯O2^ii^	0.83 (4)	2.91 (4)	3.225 (3)	105 (3)
N3—H1*N*3⋯O7^ii^	0.83 (4)	2.70 (5)	3.059 (3)	108 (3)
N3—H2*N*3⋯O7^ii^	0.74 (4)	2.69 (5)	3.059 (3)	114 (4)
O7—H1*O*7⋯O2^ii^	0.79 (4)	2.08 (4)	2.857 (3)	166 (3)

Symmetry codes: (i) 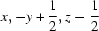; (ii) 
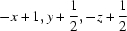
.

**Fig. 1.**
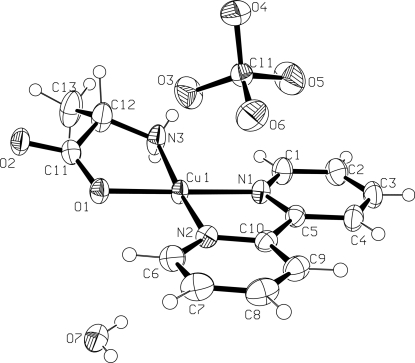
The asymmetric unit in (I) showing the crystallographic numbering scheme. Displacement ellipsoids are shown at the 50% probability level.

**Fig. 2.**
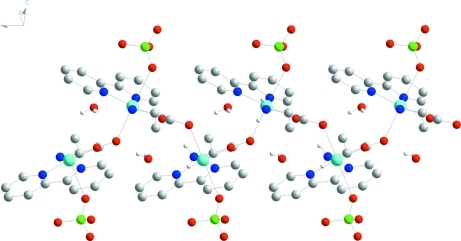
Single strand supramolecular assembly mediated by further coordination of the carboxylato group of the aminoacid. Colour code: cyan = Cu, green = Cl, red = O, blue = N, grey = C. Hydrogen atoms have been omitted for clarity.

**Fig. 3.**
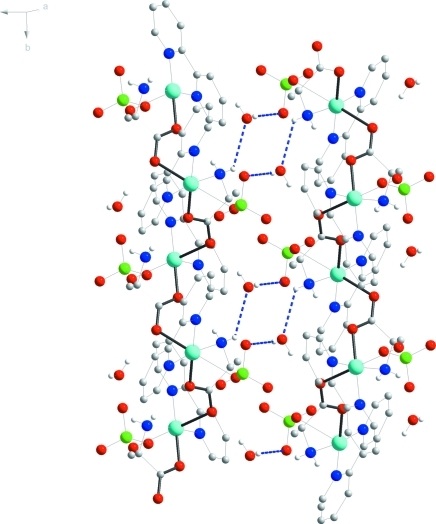
Supramolecular assembly at the secondary level formed by H-bond formation. The single strand chain is represented with thick bonds whereas the H-bonds are represented by blue dashed lines. For clarity only hydrogens (shown in white) involved in intermolecular associations are represented.

**Fig. 4.**
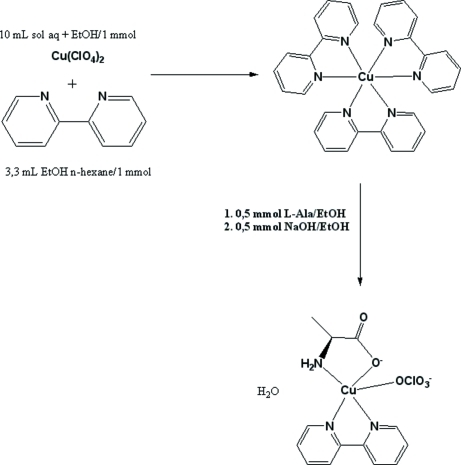
The formation of the title compound.

## supplementary crystallographic information

### Comment

The structure of the title complex, (I) and Fig. 1, is of interest with respect to the stereochemistry of the complexed aminoacid, the coordination geometry of the metal centre and the single-stranded supramolecular assembly created primarly by further coordination of the carboxylate group of the aminoacid, Fig. 2. The secondary association is by crosslinks realised through H-bonds between the created chains, Fig. 3. The supramolecular structure described for (I) is found in other (aminoacidato)(2,2'-bipyridyl)copper(II) complexes, such as in the tryptophanato (Masuda *et al.*, 1991) and aspartato complexes (Antolini *et al.*, 1983). The assembly has also been identified in the proline complex but not described as a supramolecular association (Sgarabotto *et al.*, 1999). For the alaninate complex, see also Solans *et al.* (1992).

### Experimental

The synthesis of (I) was realized by using an intermediate complex, i.e. tris(2,2'-bipyridyl)copper(II), as shown in Fig. 4. The cation in (I) was prepared according to the following procedure: Two ethanolic solutions, one containing 2,2'-bipyridyl (0.31 g, 2 mmol/5 mL) and another containing Cu(ClO_4_)_2_.2H_2_O (0.6 g, 2 mmol/5 mL) were mixed with stirring. To the resulting suspension of a blue powder, an alkaline solution of L-alanine (0.18 g alanine + 0.08 g NaOH 2 mmol/10 mL water) was added dropwise (see also Scheme 2). The suspension cleared and changed colour to dark-blue. The mixture was heated to 50°C and Na_2_ClO_4_ (1 mmol) was added. After 10 mins, the solution was cooled and filtered. The filtrate was allowed to stand at room temperature for several days when dark-blue crystals, suitable for X-ray analysis, separated, collected and washed with a methanolic solution.

### Refinement

The H atoms were refined freely: O-H = 0.69 (5) - 0.79 (3) Å, N-H = 0.73 (4) - 0.83 (3) Å, and C-H = 0.89 (3) - 1.16 (4) Å.

### Crystal data

**Table:**

[Cu(C_3_H_6_NO_2_)(C_10_H_8_N_2_)]ClO_4_·H_2_O	*F*(000) = 868
*M**_r_* = 425.28	*D*_x_ = 1.718 Mg m^−^^3^
Monoclinic, *P*2_1_/*c*	Melting point: 253 K
Hall symbol: -P 2ybc	Mo *K*α radiation, λ = 0.71073 Å
*a* = 13.1807 (10) Å	Cell parameters from 4860 reflections
*b* = 8.2656 (6) Å	θ = 2.7–28.3°
*c* = 16.1195 (13) Å	µ = 1.53 mm^−^^1^
β = 110.606 (2)°	*T* = 220 K
*V* = 1643.8 (2) Å^3^	Prism, dark blue
*Z* = 4	0.60 × 0.30 × 0.30 mm

### Data collection

**Table:**

Bruker SMART CCD area-detector diffractometer	3939 independent reflections
Radiation source: fine-focus sealed tube	3611 reflections with *I* > 2σ(*I*)
graphite	*R*_int_ = 0.020
Detector resolution: 81.92 pixels mm^-1^	θ_max_ = 28.3°, θ_min_ = 2.6°
φ scans	*h* = −17→17
Absorption correction: multi-scan (*SADABS*; Sheldrick, 1997)	*k* = −11→11
*T*_min_ = 0.460, *T*_max_ = 0.656	*l* = −20→21
13671 measured reflections	

### Refinement

**Table:**

Refinement on *F*^2^	Primary atom site location: structure-invariant direct methods
Least-squares matrix: full	Secondary atom site location: difference Fourier map
*R*[*F*^2^ > 2σ(*F*^2^)] = 0.033	Hydrogen site location: difference Fourier map
*wR*(*F*^2^) = 0.078	All H-atom parameters refined
*S* = 1.11	*w* = 1/[σ^2^(*F*_o_^2^) + (0.0317*P*)^2^ + 1.4344*P*] where *P* = (*F*_o_^2^ + 2*F*_c_^2^)/3
3939 reflections	(Δ/σ)_max_ = 0.001
290 parameters	Δρ_max_ = 0.89 e Å^−^^3^
0 restraints	Δρ_min_ = −0.43 e Å^−^^3^

### Special details

**Table:**

Geometry. All e.s.d.'s (except the e.s.d. in the dihedral angle between two l.s. planes) are estimated using the full covariance matrix. The cell e.s.d.'s are taken into account individually in the estimation of e.s.d.'s in distances, angles and torsion angles; correlations between e.s.d.'s in cell parameters are only used when they are defined by crystal symmetry. An approximate (isotropic) treatment of cell e.s.d.'s is used for estimating e.s.d.'s involving l.s. planes.
Refinement. Refinement of *F*^2^ against ALL reflections. The weighted *R*-factor *wR* and goodness of fit *S* are based on *F*^2^, conventional *R*-factors *R* are based on *F*, with *F* set to zero for negative *F*^2^. The threshold expression of *F*^2^ > σ(*F*^2^) is used only for calculating *R*-factors(gt) *etc*. and is not relevant to the choice of reflections for refinement. *R*-factors based on *F*^2^ are statistically about twice as large as those based on *F*, and *R*- factors based on ALL data will be even larger.

### Fractional atomic coordinates and isotropic or equivalent isotropic displacement parameters (Å^2^)

**Table:**

	*x*	*y*	*z*	*U*_iso_*/*U*_eq_	
Cu1	0.364703 (18)	0.28840 (3)	0.167573 (16)	0.02538 (8)	
Cl1	0.17905 (4)	0.19812 (6)	−0.06197 (3)	0.02952 (11)	
O1	0.43017 (11)	0.07522 (17)	0.20438 (10)	0.0300 (3)	
O2	0.55154 (13)	−0.09670 (18)	0.18782 (10)	0.0365 (3)	
O3	0.28355 (13)	0.1556 (3)	0.00253 (12)	0.0505 (4)	
O4	0.16505 (15)	0.1197 (2)	−0.14439 (11)	0.0450 (4)	
O5	0.17426 (19)	0.3705 (2)	−0.07423 (13)	0.0605 (5)	
O6	0.09645 (14)	0.1466 (3)	−0.02883 (12)	0.0510 (4)	
N1	0.28664 (13)	0.4972 (2)	0.12453 (11)	0.0275 (3)	
N2	0.22426 (13)	0.2334 (2)	0.18192 (11)	0.0259 (3)	
N3	0.49264 (17)	0.3247 (2)	0.13184 (19)	0.0391 (5)	
C1	0.32627 (18)	0.6292 (3)	0.09841 (16)	0.0358 (5)	
C2	0.2644 (2)	0.7669 (3)	0.06765 (17)	0.0398 (5)	
C3	0.1579 (2)	0.7682 (3)	0.06305 (16)	0.0382 (5)	
C4	0.11629 (18)	0.6331 (3)	0.09049 (15)	0.0350 (5)	
C5	0.18218 (15)	0.4985 (2)	0.12098 (12)	0.0264 (4)	
C6	0.19879 (18)	0.0907 (3)	0.20930 (14)	0.0330 (4)	
C7	0.0946 (2)	0.0558 (3)	0.20633 (16)	0.0391 (5)	
C8	0.01505 (19)	0.1715 (3)	0.17510 (16)	0.0391 (5)	
C9	0.04044 (17)	0.3198 (3)	0.14800 (14)	0.0340 (4)	
C10	0.14634 (15)	0.3474 (2)	0.15143 (12)	0.0264 (4)	
C11	0.50804 (16)	0.0390 (2)	0.17798 (13)	0.0288 (4)	
C12	0.5466 (2)	0.1693 (3)	0.12787 (19)	0.0429 (5)	
C13	0.6666 (2)	0.1814 (4)	0.1552 (3)	0.0605 (8)	
H2N3	0.482 (4)	0.357 (6)	0.087 (3)	0.098 (16)*	
H1N3	0.536 (3)	0.384 (5)	0.170 (3)	0.083 (13)*	
H1	0.398 (2)	0.628 (3)	0.1013 (17)	0.042 (7)*	
H2	0.293 (2)	0.844 (4)	0.0488 (19)	0.048 (8)*	
H3	0.116 (2)	0.855 (4)	0.0419 (17)	0.044 (7)*	
H4	0.049 (2)	0.634 (4)	0.0873 (19)	0.054 (8)*	
H6	0.257 (2)	0.014 (3)	0.2313 (16)	0.037 (6)*	
H7	0.082 (2)	−0.043 (4)	0.2244 (18)	0.045 (7)*	
H8	−0.057 (2)	0.151 (4)	0.1713 (18)	0.052 (8)*	
H9	−0.014 (2)	0.401 (4)	0.1242 (18)	0.046 (7)*	
H12	0.539 (3)	0.124 (5)	0.059 (3)	0.094 (12)*	
H13A	0.691 (3)	0.261 (4)	0.116 (2)	0.073 (10)*	
H13B	0.699 (3)	0.072 (5)	0.145 (2)	0.077 (11)*	
H13C	0.691 (4)	0.225 (5)	0.228 (3)	0.105 (15)*	
O7	0.4174 (2)	0.1391 (3)	0.41504 (15)	0.0516 (5)	
H1O7	0.418 (3)	0.220 (4)	0.389 (2)	0.056 (10)*	
H2O7	0.367 (4)	0.142 (7)	0.419 (3)	0.110 (19)*	

### Atomic displacement parameters (Å^2^)

**Table:**

	*U*^11^	*U*^22^	*U*^33^	*U*^12^	*U*^13^	*U*^23^
Cu1	0.02196 (12)	0.01912 (12)	0.03468 (14)	0.00188 (8)	0.00948 (9)	0.00357 (9)
Cl1	0.0286 (2)	0.0299 (2)	0.0284 (2)	0.00289 (17)	0.00796 (18)	0.00106 (18)
O1	0.0300 (7)	0.0218 (7)	0.0391 (8)	0.0031 (5)	0.0133 (6)	0.0050 (6)
O2	0.0399 (8)	0.0251 (7)	0.0431 (8)	0.0101 (6)	0.0128 (7)	0.0037 (6)
O3	0.0284 (8)	0.0680 (13)	0.0458 (9)	0.0050 (8)	0.0016 (7)	0.0056 (9)
O4	0.0561 (10)	0.0443 (10)	0.0355 (8)	0.0040 (8)	0.0171 (7)	−0.0061 (7)
O5	0.0954 (16)	0.0274 (9)	0.0515 (11)	0.0022 (9)	0.0167 (10)	0.0023 (8)
O6	0.0375 (9)	0.0659 (13)	0.0562 (11)	0.0028 (8)	0.0248 (8)	0.0028 (9)
N1	0.0251 (8)	0.0227 (8)	0.0333 (8)	0.0019 (6)	0.0082 (6)	0.0011 (6)
N2	0.0253 (8)	0.0245 (8)	0.0276 (8)	−0.0015 (6)	0.0088 (6)	−0.0017 (6)
N3	0.0310 (10)	0.0249 (9)	0.0666 (15)	0.0059 (7)	0.0237 (10)	0.0115 (10)
C1	0.0320 (11)	0.0268 (10)	0.0480 (12)	0.0003 (8)	0.0132 (9)	0.0051 (9)
C2	0.0467 (13)	0.0245 (10)	0.0470 (13)	0.0005 (9)	0.0149 (10)	0.0051 (9)
C3	0.0416 (12)	0.0288 (11)	0.0378 (11)	0.0118 (9)	0.0062 (9)	0.0029 (9)
C4	0.0287 (10)	0.0347 (11)	0.0372 (11)	0.0091 (9)	0.0060 (8)	−0.0004 (9)
C5	0.0245 (9)	0.0263 (9)	0.0257 (9)	0.0023 (7)	0.0054 (7)	−0.0027 (7)
C6	0.0356 (11)	0.0284 (10)	0.0353 (10)	−0.0028 (8)	0.0131 (9)	−0.0010 (8)
C7	0.0436 (12)	0.0347 (12)	0.0442 (12)	−0.0134 (10)	0.0220 (10)	−0.0047 (10)
C8	0.0299 (10)	0.0483 (13)	0.0425 (12)	−0.0106 (10)	0.0168 (9)	−0.0093 (10)
C9	0.0254 (9)	0.0426 (12)	0.0337 (10)	−0.0007 (9)	0.0098 (8)	−0.0057 (9)
C10	0.0248 (9)	0.0289 (10)	0.0245 (8)	−0.0008 (7)	0.0074 (7)	−0.0049 (7)
C11	0.0261 (9)	0.0247 (9)	0.0309 (9)	0.0011 (7)	0.0042 (7)	0.0014 (8)
C12	0.0388 (12)	0.0332 (12)	0.0623 (15)	0.0089 (9)	0.0249 (11)	0.0115 (11)
C13	0.0427 (14)	0.0452 (15)	0.105 (3)	0.0125 (12)	0.0408 (16)	0.0190 (16)
O7	0.0602 (13)	0.0449 (11)	0.0585 (12)	0.0192 (9)	0.0318 (10)	0.0107 (9)

### Geometric parameters (Å, °)

**Table:**

Cu1—O1	1.9598 (14)	C2—H2	0.85 (3)
Cu1—N3	1.987 (2)	C3—C4	1.384 (4)
Cu1—N2	1.9970 (16)	C3—H3	0.90 (3)
Cu1—N1	2.0043 (17)	C4—C5	1.390 (3)
Cu1—O2^i^	2.3965 (16)	C4—H4	0.86 (3)
Cl1—O4	1.4307 (16)	C5—C10	1.479 (3)
Cl1—O6	1.4358 (18)	C6—C7	1.387 (3)
Cl1—O5	1.4365 (19)	C6—H6	0.96 (3)
Cl1—O3	1.4469 (16)	C7—C8	1.377 (4)
O1—C11	1.277 (2)	C7—H7	0.90 (3)
O2—C11	1.244 (2)	C8—C9	1.381 (3)
O2—Cu1^ii^	2.3965 (16)	C8—H8	0.95 (3)
N1—C1	1.340 (3)	C9—C10	1.396 (3)
N1—C5	1.358 (2)	C9—H9	0.96 (3)
N2—C6	1.343 (3)	C11—C12	1.536 (3)
N2—C10	1.353 (3)	C12—C13	1.488 (4)
N3—C12	1.481 (3)	C12—H12	1.15 (4)
N3—H2N3	0.74 (4)	C13—H13A	1.04 (4)
N3—H1N3	0.83 (4)	C13—H13B	1.04 (4)
C1—C2	1.386 (3)	C13—H13C	1.16 (5)
C1—H1	0.92 (3)	O7—H1O7	0.79 (4)
C2—C3	1.380 (4)	O7—H2O7	0.69 (5)
			
O1—Cu1—N3	83.99 (7)	C4—C3—H3	120.4 (18)
O1—Cu1—N2	95.03 (6)	C3—C4—C5	119.4 (2)
N3—Cu1—N2	169.62 (10)	C3—C4—H4	119 (2)
O1—Cu1—N1	175.41 (6)	C5—C4—H4	122 (2)
N3—Cu1—N1	98.87 (7)	N1—C5—C4	121.28 (19)
N2—Cu1—N1	81.51 (7)	N1—C5—C10	114.65 (16)
O1—Cu1—O2^i^	93.37 (6)	C4—C5—C10	124.07 (19)
N3—Cu1—O2^i^	94.28 (9)	N2—C6—C7	122.0 (2)
N2—Cu1—O2^i^	96.09 (6)	N2—C6—H6	116.2 (16)
N1—Cu1—O2^i^	90.00 (6)	C7—C6—H6	121.8 (16)
O4—Cl1—O6	110.13 (11)	C8—C7—C6	119.0 (2)
O4—Cl1—O5	109.66 (11)	C8—C7—H7	123.1 (18)
O6—Cl1—O5	110.07 (13)	C6—C7—H7	117.9 (18)
O4—Cl1—O3	109.65 (11)	C7—C8—C9	119.6 (2)
O6—Cl1—O3	108.38 (11)	C7—C8—H8	121.0 (19)
O5—Cl1—O3	108.93 (13)	C9—C8—H8	119.4 (19)
C11—O1—Cu1	115.46 (12)	C8—C9—C10	118.9 (2)
C11—O2—Cu1^ii^	121.01 (14)	C8—C9—H9	120.8 (17)
C1—N1—C5	118.82 (17)	C10—C9—H9	120.3 (17)
C1—N1—Cu1	126.92 (14)	N2—C10—C9	121.42 (19)
C5—N1—Cu1	114.25 (13)	N2—C10—C5	114.75 (16)
C6—N2—C10	119.06 (18)	C9—C10—C5	123.82 (19)
C6—N2—Cu1	125.95 (14)	O2—C11—O1	123.87 (19)
C10—N2—Cu1	114.56 (13)	O2—C11—C12	118.42 (19)
C12—N3—Cu1	110.57 (14)	O1—C11—C12	117.66 (18)
C12—N3—H2N3	101 (4)	N3—C12—C13	113.9 (2)
Cu1—N3—H2N3	117 (3)	N3—C12—C11	109.41 (19)
C12—N3—H1N3	109 (3)	C13—C12—C11	113.9 (2)
Cu1—N3—H1N3	108 (3)	N3—C12—H12	116 (2)
H2N3—N3—H1N3	111 (4)	C13—C12—H12	91.9 (19)
N1—C1—C2	122.4 (2)	C11—C12—H12	111 (2)
N1—C1—H1	118.5 (18)	C12—C13—H13A	113 (2)
C2—C1—H1	119.1 (18)	C12—C13—H13B	111 (2)
C3—C2—C1	119.0 (2)	H13A—C13—H13B	102 (3)
C3—C2—H2	123 (2)	C12—C13—H13C	102 (2)
C1—C2—H2	118 (2)	H13A—C13—H13C	113 (3)
C2—C3—C4	119.1 (2)	H13B—C13—H13C	117 (3)
C2—C3—H3	120.5 (18)	H1O7—O7—H2O7	102 (5)

Symmetry codes: (i) −*x*+1, *y*+1/2, −*z*+1/2; (ii) −*x*+1, *y*−1/2, −*z*+1/2.

### Hydrogen-bond geometry (Å, °)

**Table:**

*D*—H···*A*	*D*—H	H···*A*	*D*···*A*	*D*—H···*A*
N3—H2N3···O7^iii^	0.74 (4)	2.60 (4)	3.293 (4)	159 (5)
N3—H1N3···O1^i^	0.83 (4)	2.48 (4)	3.225 (3)	149 (3)
N3—H1N3···O2^i^	0.83 (4)	2.91 (4)	3.225 (3)	105 (3)
N3—H1N3···O7^i^	0.83 (4)	2.70 (5)	3.059 (3)	108 (3)
N3—H2N3···O7^i^	0.74 (4)	2.69 (5)	3.059 (3)	114 (4)
O7—H1O7···O2^i^	0.79 (4)	2.08 (4)	2.857 (3)	166 (3)

Symmetry codes: (iii) *x*, −*y*+1/2, *z*−1/2; (i) −*x*+1, *y*+1/2, −*z*+1/2.
